# Uncovering knowledge of pediatric sepsis and recognition of septic shock: a survey among Brazilian pediatricians

**DOI:** 10.62675/2965-2774.20250143

**Published:** 2025-03-17

**Authors:** Andrea Maria Cordeiro Ventura, Orlei Ribeiro Araujo, José Colleti, Daniela Carla de Souza

**Affiliations:** 1 Universidade de São Paulo Hospital Universitário São Paulo SP Brazil Hospital Universitário, Universidade de São Paulo - São Paulo (SP), Brazil.; 2 Universidade Federal de São Paulo Instituto de Oncologia Pediátrica Grupo de Apoio ao Adolescente e à Criança com Câncer São Paulo SP Brazil Grupo de Apoio ao Adolescente e à Criança com Câncer, Instituto de Oncologia Pediátrica, Universidade Federal de São Paulo - São Paulo (SP), Brazil.; 3 Hospital Israelita Albert Einstein Department of Pediatrics São Paulo SP Brazil Department of Pediatrics, Hospital Israelita Albert Einstein - São Paulo (SP), Brazil.; 4 Universidade de São Paulo Hospital Universitário Pediatric Intensive Care Unit São Paulo SP Brazil Pediatric Intensive Care Unit, Hospital Universitário, Universidade de São Paulo - São Paulo (SP), Brazil.

**Keywords:** Sepsis, Septic shock, Child, Evidence gaps, Survey and questionnaires, Brazil

## Abstract

**Objective:**

To evaluate the ability of Brazilian pediatricians to recognize sepsis and septic shock in children.

**Methods:**

A cross-sectional multicenter survey was conducted among Brazilian pediatricians between May and June 2023, before the release of the new Phoenix sepsis criteria in 2024. An online electronic system was used for surveys to characterize the knowledge and skills of physicians in the diagnosis and treatment of sepsis in children. The questions related to the diagnosis and treatment of sepsis and septic shock in children were based on the International Pediatric Sepsis Consensus Conference, the American College of Critical Care Medicine Guidelines, and the Surviving Sepsis Campaign in Pediatrics. Descriptive statistical analyses were performed using R software.

**Results:**

Pediatricians were surveyed about the recognition, knowledge, and management of pediatric patients with sepsis and septic shock. The responses of 355 physicians from all regions of Brazil were analyzed, with the majority from the southeastern region of the country (53.3%). In clinical practice, the most utilized criteria for diagnosing sepsis included inappropriate tachycardia (92%), temperature alteration (88.2%), and the presence of a suspected or confirmed infectious focus (87.9%). For septic shock, capillary refill time alteration (87.1%), arterial hypotension (84.8%), and changes in the level of consciousness (82.2%) were the predominant indicators. A total of 55.6% pediatricians reported having the potential to obtain venous or intraosseous access within 5 minutes, and 59.3% could administer antibiotics within the first hour. Approximately one-quarter (27.5%) of the participants responded that it was possible to infuse 40 - 60mL/kg in 1 hour. The most commonly used solution for fluid resuscitation was isotonic saline (70.9%), followed by Ringer's lactate (45.0%). The infusion of a vasopressor was considered in patients who did not improve after receiving an infusion of 40 - 60mL/kg (75.8%).

**Conclusion:**

This is the first study to assess the knowledge of sepsis and septic shock among the Brazilian pediatric population. The results reveal a significant deficiency in the recognition of sepsis. This study demonstrated a gap between evidence and clinical practice. Adherence to international guidelines needs to be improved.

## INTRODUCTION

Sepsis is a potentially life-threatening condition that occurs when the host's response to infection leads to the dysfunction of multiple organs and systems.^( 1 )^ In 2017, the World Health Organization recognized sepsis as a public health priority and began to demand the adoption of measures for sepsis prevention, diagnosis, and treatment.^( 2 )^ These measures aim to improve the prognosis of sepsis patients, especially in settings with limited resources, where the burden of the disease appears to be greater.^( 3 )^

To improve sepsis recognition, treatment, and outcomes, some guidelines have been published. The main guidelines include the International Pediatric Sepsis Consensus Conference (IPSCC),^( 1 )^ the American College of Critical Care Medicine Guidelines (ACCCMG),^( 4 )^ and the Surviving Sepsis Campaign (SSC) in Pediatrics.^( 5 )^ These guidelines are mainly based on expert opinions regarding the recognition, classification, and treatment of pediatric patients with sepsis. The systemic inflammatory response to syndrome (SIRS) and the presence of or suspicion of an infection played an important role in the development of those guidelines, along with treatment bundles to guide management. The goal was to improve the sensitivity of sepsis recognition and improve outcomes.

Barriers related to delays in diagnosis and treatment are linked to the characteristics of sepsis itself, difficulties in defining sepsis and diagnosing infection, lack of knowledge about sepsis, attitudes, and behaviors toward sepsis and guidelines, as well as the lack of effective resources and processes.^( 6 )^ All of these factors contribute to discrepancies between scientific evidence and clinical practice.^( 7 )^ Recognizing the importance of adhering to current guidelines, especially in the care of children with sepsis, we embarked on a study to assess the proficiency of Brazilian pediatricians in identifying and managing sepsis and septic shock in pediatric patients.

## METHODS

A prospective, observational, multicenter, descriptive study was conducted. An online electronic system, Research Electronic Data Capture (REDCap), was used for surveys to characterize the knowledge and skills of physicians in the diagnosis and treatment of sepsis in children. The study was approved by the Institutional Review Board of the *Hospital Universitário* of *Universidade de São Paulo* .

The questionnaire was developed and validated with the participation of the Brazilian Research Network in Pediatric Intensive Care (BRnet-PIC). The questionnaire was developed before the new Phoenix criteria were released and included questions to characterize the participants, knowledge of the diagnosis and treatment of sepsis in children, and clinical scenarios. It was sent by email by the Brazilian Society of Pediatrics on 3 different occasions with 15-day intervals between May and June 2023, to pediatricians from various specialties practicing in all regions of Brazil.

The results of the descriptive statistical analyses are expressed as numbers and percentages. A significance level of p < 0.05 was considered statistically significant. Chi-square tests were used for comparisons between groups. The data analysis was conducted via R statistical software (v4.1.2; R Core Team 2021).

## RESULTS

The responses of 355 physicians from all regions of Brazil were analyzed, with the majority from the southeastern region of the country (53.3%). Most participants were female, with a median age of 42 years ( Table 1S - Supplementary Material ).

Table 1 presents the data regarding self-reported knowledge of guidelines for the diagnosis and treatment of sepsis in children according to the pediatric specialty of the participant. The best-known guidelines are IPSCC, followed by SSC.

**Table 1:** Knowledge of sepsis diagnosis and treatment guidelines in children

Specialty	Responses	Percentage of knowledge of each guideline				
		IPSCC, 2005	ACCM, 2017	SSC, 2020	Other	None
General pediatrician	228	80.3	55.7	66.2	6.1	11.0
Emergency care clinician	27	92.6	66.7	85.2	14.8	0
Pediatric intensivist	100	88.0	60.0	84.0	10.0	4.0
Neonatologist	48	93.8	54.2	70.8	6.3	4.2
Resident (Pediatrics/Intensive care)	57	86.0	45.6	87.7	0	0
Other pediatric specialties	64	73.4	57.8	65.6	9.4	4.7

IPSCC - International Pediatric Sepsis Consensus Conference; ACCM - American College of Critical Care Medicine Guidelines; SSC -Surviving Sepsis Campaign.

**Figure 1:**
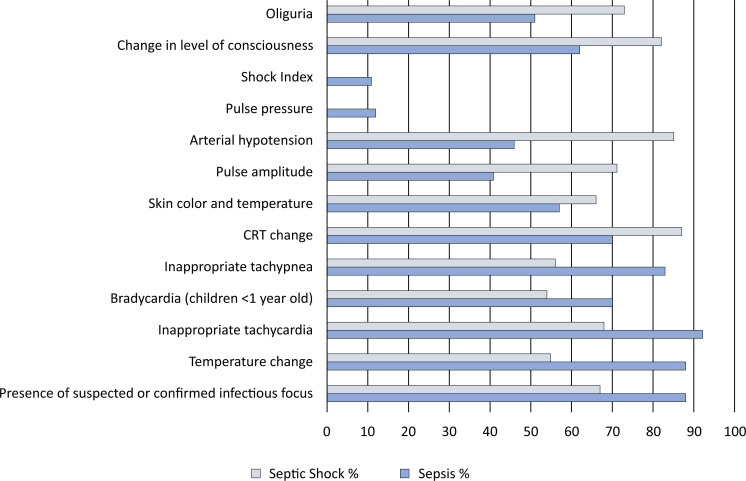
Criteria used to diagnose sepsis and septic shock.

We asked about the main criteria used to diagnose sepsis in clinical practice given that the main guidelines before the Phoenix sepsis criteria were released were based on the presence of the SIRS criteria and an infection. The physicians reported that the most frequently used criterion for the diagnosis of sepsis was inappropriate tachycardia (92%), followed by temperature alteration (88.2%). However, for septic shock, capillary refill time alteration was the most frequently used criterion (87.1%), followed by arterial hypotension (84.8%) ( Figure 1 ). We also sought to determine whether nonobjective criteria were used to diagnose sepsis. Most participants (64%) considered the opinions of family members for the diagnosis of sepsis, whereas 80.2% always valued their own intuition ("gut feeling") to diagnose sepsis.

CRT - capillary refill time.

Regarding alerts for sepsis diagnosis, 54.2% reported that clinical suspicion was raised by the medical team, 27.4% by nursing, and 34.6% by any member of the multiprofessional team.

The Surviving Sepsis Campaign guidelines suggest starting antibiotics within 1 hour of septic shock recognition; 55.6% of respondents reported the possibility of obtaining venous or intraosseous access within 5 minutes; 70.2% had resources for culture collection; and 59.3% could administer antibiotics within the first hour. One-third (32.4%) of the participants reported that they could administer antibiotics within the first hour but were unsure if the time was monitored.

In table 2S (Supplementary Material) , we present the data regarding the resuscitation volume rates in different scenarios. Approximately one-quarter (27.5%) of the participants said it was possible to infuse 40 - 60mL/kg in 1 hour, which is consistent with international guidelines. The most commonly used solution for fluid resuscitation was isotonic saline (70.9%), followed by Ringer's lactate (45.0%).

The infusion of a vasopressor in patients who do not improve after receiving an infusion of 40 - 60mL/kg is recommended by the guidelines, which was considered by 75.8% of the respondents. In hypotensive patients or those with signs of hypoperfusion, drug administration was deemed necessary, regardless of the volume received (27.7%).

Most participants monitored patient response through the evaluation of clinical signs such as capillary refill time (93.6%), pulse characteristics (85.3%), and skin color and temperature (86.1%). A low percentage of participants reported the use of invasive blood pressure (3.2%), central venous pressure (18.5%), and continuous monitoring of central venous oxygen saturation (18.5%). Monitoring of central venous oxygen saturation was reported as feasible in the only intensive care unit (ICU) (48.7%), in the emergency department (13.2%), continuously (4.3%) or intermittently (21.3%).

In the clinical scenarios ( Table 3S - Supplementary Material ), the percentages of physicians who correctly recognized SIRS, sepsis, severe sepsis, septic shock, and uncomplicated infection were 52.6%, 31.3%, 30%, 77%, and 80.2%, respectively. The correct answers by institution are reported in table 4S (Supplementary Material) , and all the clinical scenarios are described in table 5S (Supplementary Material) .

## DISCUSSION

To our knowledge, this study represents the first attempt to delineate the knowledge profile of Brazilian pediatricians across various specialties regarding the diagnosis and treatment of sepsis and septic shock in children. The findings revealed a notable deficit in overall knowledge, particularly regarding the diagnosis and treatment guidelines established by professional bodies.

A significant number of studies have reported definitions of pediatric sepsis since 2005,^( 1 )^ but overall knowledge about the treatment guidelines proposed by the ACCM/PALS in 2017 and the CSS in 2020 has been low.^( 4 , 5 )^ The analysis of responses to clinical scenarios suggests a higher rate of correct answers at the extremes of the spectrum, with uncomplicated infection and shock having the highest rates of accuracy.

We observed that the clinical criteria for SIRS were highly valuable for the diagnosis of sepsis and septic shock. This study indicates an overlap in the clinical criteria used to diagnose sepsis and septic shock, which has already been demonstrated in previous studies that reported discrepancies in how the IPSCC^( 1 )^ criteria are applied clinically, which limits the precise characterization of sepsis and septic shock. Our results also indicate that Brazilian pediatricians perceive sepsis as a potentially life-threatening organic dysfunction within the context of infection. This understanding is underscored by the fact that a significant percentage of physicians value clinical criteria for diagnosing sepsis, such as changes in the level of consciousness, capillary refill time, skin color and temperature, and oliguria. This perception is consistent with the new Phoenix criteria for sepsis.

The evaluation of aspects related to treatment revealed low adherence to current guidelines (e.g., low antibiotic infusion rate in the first hour, lack of control of time until antibiotic infusion, and low use of vasoactive/inotropic agents). Difficulties in global adherence to treatment guidelines are related to technical and/or human resource limitations, such as a lack of skill with certain procedures and medication management and difficulties in obtaining peripheral, intraosseous, or central venous access.^( 8 )^ These aspects of low adherence to international guidelines could alter patient outcomes, highlighting that it is important to have knowledge of the guidelines but also to have resources to implement them.

Our results revealed a low percentage of fluid infusion in the first hour. The SSC guidelines suggest administering up to 40 - 60mL/kg in bolus fluid (10 - 20mL/kg per bolus) over the first hour. Refractory shock to fluids was considered by the majority when there was no improvement after infusion of 40 - 60mL/kg; however, the time for infusion of this volume was not controlled in most responses. We observed that monitoring was essentially performed on the basis of clinical data, with a minority of responses reporting the use of lactate, SvcO_2_, or even minimally invasive methods of cardiac output measurements. The SSC guidelines suggest the use of advanced hemodynamic variables, when available, in addition to bedside clinical variables, to guide the resuscitation of children with septic shock or other sepsis-associated organ dysfunction.

The results of this research confirmed those of studies conducted in other countries.^( 9 – 11 )^

Assunção et al. evaluated the knowledge of clinicians about sepsis and concluded that the recognition of sepsis and its severity is unsatisfactory.^( 9 )^ In Latin America, a sepsis working group evaluated the self-reported adherence to the diagnostic and treatment guidelines of sepsis from the ACCM in 2017 among 898 pediatric emergency physicians (15 Brazilians) from 14 countries. The participants reported that early recognition of sepsis (53%) and timely infusion of inotropic agents (39%) were the main barriers to the diagnosis and treatment of children with sepsis.^( 10 )^ Regina et al. conducted a survey among physicians, nurses and paramedics at a tertiary Swiss medical center and reported a deficit in sepsis awareness and knowledge, reflecting a lack of sepsis-specific continuing education requiring immediate corrective measures.^( 12 )^

In January 2024, the International Consensus Criteria for Pediatric Sepsis and Septic Shock (Phoenix Sepsis Criteria) were published in JAMA.^( 13 )^ The Phoenix Sepsis Criteria were derived and validated on the basis of three sequential and coordinated studies (global survey, systematic review/meta-analysis, and a large international database). Whether the implementation of the new criteria in routine clinical settings will enhance the identification of children with sepsis and septic shock, leading to prompt intervention and a decrease in the morbidity and mortality rates associated with pediatric sepsis, remains to be determined. Hence, the new pediatric sepsis criteria must be prospectively validated.

Considering the limitations of this study, it is crucial to acknowledge the potential for selection bias, as participants who consent to participate may not comprehensively reflect the broader population. The respondents might possess a particular interest or expertise in the subject matter, thus affecting the representativeness of the sample. Moreover, the findings may not be universally applicable to all pediatricians because of variations in geographic location, practice settings, and levels of experience. Notably, this study revealed a notable skew toward participants from the southeastern region of the country. Furthermore, the self-reported nature of survey data introduces the possibility of response inaccuracies, stemming from factors such as misinterpretation, challenges in recalling real-life behaviors, and the influence of social desirability bias.

## CONCLUSION

In conclusion, Brazilian pediatricians exhibit deficiencies in recognizing and managing pediatric sepsis, warranting urgent attention to enhance adherence to international guidelines. Our findings underscore the imperative for targeted educational initiatives aimed at closing the gap between evidence-based recommendations and clinical practice, particularly in resource-constrained settings.
