# Supplementary material for: Uncovering knowledge of pediatric sepsis and recognition of septic shock: a survey among Brazilian pediatricians
Source: Crit Care Sci. 2025 Mar 17;37:e20250143. doi: 10.62675/2965-2774.20250143 (PMC12133085; doi:10.62675/2965-2774.20250143)
Supplement: Supplementary file 1 [file 2965-2774-ccsci-37-e20250143-Suppl01.pdf]

Critical Care Science
https://doi.org/10.62675/2965-2774.20250143

SUPPLEMENTARY MATERIAL

Uncovering knowledge of pediatric sepsis and recognition of septic shock: a survey among Brazilian pediatricians

Andrea Maria Cordeiro Ventura[1], Orlei Ribeiro Araujo[2], José Colleti Junior[3], Daniela Carla de Souza[4]

Table 1S - Demographic characteristics of participants

Participants	
Women	276 (75.2)
Age (years)	42 (33 - 54.2)
Time since graduation (years)	17 (7 - 29.5)
Time working in Pediatrics (years)	14 (4 - 27)
Type of institution	
Public	222 (62.9)
Private	101 (28.6)
University	163 (46.2)
Other	10 (2.8)

Results expressed as n (%) or median (interquartile range)

Table 2S - Resuscitation volume aliquots used according to participants

Tax rate volume	%
20mL/kg no weight limit	52.3
20mL/kg, maximum 500mL	32.8
10mL/kg no weight limit	11.3
10mL/kg, maximum 250mL	6.4

Table 3S - Percentages of correct answers for clinical scenarios, by specialties

Clinical scenario	General pediatrician (n= 228)	Pediatric intensivist (n = 100)	Neonatologist (n = 48)	Emergencist (n = 27)	Other (n = 64)	Residents (n= 60)	Average accuracy per diagnosis	p value
SIRS	51.8	68.0	54.2	33.3	53.1	55.0	52.6	0.02*
Severe sepsis	31.6	37.0	25.0	29.6	28.1	36.7	31.3	0.64†
Sepsis	27.2	41.0	18.8	29.6	23.4	40.0	30.0	0.018†
Septic shock	77.2	80.0	81.3	66.7	68.8	88.3	77.0	0.09†
Uncomplicated infection	79.0	82.0	83.3	77.0	71.9	88.3	80.2	0.3†
Average accuracy per specialty	53.4	61.6	52.5	47.2	49.1	66.7		0.0002†

SIRS - systemic inflammatory response to syndrome. * p value in the chi-square test for the proportions of correct answers per diagnosis, comparing specialties; † p value in chi-square for the proportions of total correct answers by specialty.

This is an open access article under the CC BY license (https://creativecommons.org/licenses/by/4.0/).

Table 4S - Percentages of correct answers for clinical cases, by type of institution

Clinical scenario	Public (n = 222)	University (n = 101)	Private (n = 163)	Average % of correct answers per diagnosis	p value
SIRS	58.1	56.4	54.0	56.2	0.72*
Severe sepsis	38.7	32.7	26.4	32.6	0.04*
Sepsis	37.4	29.7	29.4	32.2	0.18*
Septic shock	82.4	75.2	81.6	79.8	0.29*
Non-complicated infection	83.8	77.2	84.7	81.9	0.25*
Average % of success by type of hospital	60.1	54.3	55.2		0.01†

SIRS - systemic inflammatory response to syndrome. * p value in the chi-square test for the proportions of correct answers per diagnosis, comparing the types of service; † comparison of the proportions of total success rates by type of service.

Table 5S - Clinical scenario

Clinical scenario	
SIRS	A 10-year-old patient undergoing post-operative spinal arthrodesis. He is febrile (37.9C), HR of 140bmp, broad pulses, warm extremities and capillary refill time < 2 seconds. The BP is 110/50 (70) mmHg, and the RR is 28ipm. Diuresis= 1.5mL/kg/hour. Leukogram = 5,800 cells/mm^3. What is the most likely diagnosis?
Sepsis	A 6-year-old boy with a 3-day history of fever, cough and fatigue. It appears active, Tax = 38C, HR 144bpm, BP 100 x 50 (65) mmHg, adequate central and peripheral pulses, capillary refill time 2 seconds. RR of 55 ipm with oxygen saturation of 96% in room air, with crackling rales in the left lung. Which are the most probable diagnostics?
Severe sepsis	Girl aged 2 years and 6 months hospitalized with a diagnosis of pneumonia. She is prostrate, febrile (Tax= 38.7C), HR 162bpm, BP 83x44 (57) mmHg, adequate central pulses and fine peripheral pulses, capillary refill time of 4 seconds, RR of 62ipm, oxygen saturation of 92% at 50% Venturia mask. Leukocytosis with more than 10% of young forms. Which are the most probable diagnostics?
Septic shock	A 7-year-old boy, with fever, abdominal pain in the right iliac fossa and vomiting for 3 days, without diuresis for 12 hours. He appears prostrate, with dry mucous membranes, febrile, tachycardic, normotensive. He has adequate central pulses and thin peripheral pulses, capillary refill time of 4 seconds, tachypneic. Abdominal ultrasound suggests acute appendicitis. Initial treatment with 0.9% saline solution at 20mL/kg was started. After three expansions (60mL/kg), HR is 166bpm, BP is 80/47 (58) mmHg, capillary refill time is 5 seconds, without diuresis. Which are the most probable diagnostics?
Uncomplicated infection	15-year-old girl diagnosed with dysuria. Presents with Tax = 36.7C, HR 88bpm, adequate central and peripheral pulses, capillary refill time of 2 seconds, BP 110 x 65 (80)mmHg, eupneic. Blood leukocytes count of 12,000 cells/mm3 (no young forms) and presence of leukocyturia. Which are the most probable diagnostics?

HR - heart rate; BP - blood pressure; RR - respiratory rate. Uncovering knowledge of pediatric sepsis and recognition of septic shock: a survey among Brazilian pediatricians

QUESTIONNAIRE

How were you contacted to participate in the research?

- o Social media
- o Messages via WhatsApp
- o Forwarding by friends
- o Invitation email from the *Sociedade Brasileira de Pediatria*
- o Others

DEMOGRAPHIC DATA

- o Date of completion: ___/___/_____
- o Date of birth: ___/___/_____
- o Age: _____
- o Gender:
    - Male
    - Female
    - I prefer not to say
- o State where you currently work
    - Acre
    - Alagoas
    - Amapá
    - Amazonas
    - Bahia
    - Ceará
    - Distrito Federal
    - Holy Spirit
    - Goiás
    - Maranhão
    - Mato Grosso
    - Mato Grosso do Sul
    - Minas Gerais
    - Pará
    - Paraíba
    - Paraná
    - Pernambuco
    - Piauí
    - Rio de Janeiro
    - Rio Grande do Norte
    - Rio Grande do Sul
    - Rondônia
    - Roraima
    - Santa Catarina
    - São Paulo
    - Sergipe
    - Tocantins

MEDICAL TRAINING

- Graduation year: ___ / ___ / _____
- Time since graduation: _____
- Year of completion of Pediatrics: ___ / ___ / _____
- Length of time practicing Pediatrics: _____
- Medical specialty:
    - General pediatrics
    - Pediatric intensive care
    - Neonatology
    - Pediatric emergency
    - Other pediatric specialty
    - Other non-pediatric specialty
    - Pediatrics Resident 1st year
    - Pediatrics Resident 2nd year
    - Pediatrics Resident 3rd year
    - Pediatrics ICU Resident 1st year
    - Pediatrics ICU Resident 2nd year
    - Resident in another pediatric specialty
    - Resident in another non-pediatric specialty

WORKPLACE

- Place where you do most of your work:
    - Basic Health Unit
    - Emergency Room
    - Pediatric ICU
    - Neonatal ICU
    - Mixed ICU
    - Specialized ICU - oncology, cardiology, trauma, burns
    - Office
    - Ward/Inpatient unit
    - Transport service
    - Other

- What type of institution do you predominantly work in?
    - Public institution
    - University institution
    - Private institution
    - Other

CHARACTERIZATION VARIABLES OF KNOWLEDGE AND SKILLS IN THE DIAGNOSIS AND TREATMENT OF CHILDREN WITH SEPSIS AND SEPTIC SHOCK

- Are you familiar with the definitions of SEPSIS/SEPTIC SHOCK proposed by the 2005 “International Pediatric Sepsis Consensus Conference”?
    - No
    - Yes

- What guidelines for the diagnosis and treatment of SEPSIS/SEPTIC SHOCK do you know?
  - I am not aware of any pediatric sepsis treatment guidelines.
  - I am aware of the ACCM/PALS 2017 treatment guidelines
  - I am familiar with the guidelines of the *"Surviving Sepsis Campaign"* 2020
  - I know other guidelines

- When diagnosing SEPSIS in children, do you take the opinion of family members into account?
  - No
  - Yes

- What opinion do you take into account from your family?
  - Something is wrong, he/she is not like that
  - This disease is different from others he/she has had.
  - My child has a different cry
  - My child is very sleepy
  - I'm worried about the fever
  - Other

- In your clinical practice, what clinical criteria do you use to make an early diagnosis of SEPSIS?
  - Presence of suspected or confirmed focus
  - Change in temperature (hypothermia or fever)
  - Inappropriate tachycardia
  - Bradycardia for children < 1 year
  - Inappropriate tachypnea
  - Change in capillary refill time
  - Skin color and temperature
  - Pulse amplitude
  - Change in diuresis
  - Change in level of consciousness
  - Hypotension
  - Pulse pressure (SBP minus DBP)
  - Shock index (SBP divided by HR)
  - Other

- Do you value your clinical impression (GUT feeling) to think about SEPSIS/SEPTIC SHOCK?
  - Never
  - Sometimes
  - Always

- If you answered YES to the previous question, what parameters do you use in your clinical impression (GUT feeling) to think about SEPSIS/SEPTIC SHOCK? Select more than one alternative, if necessary.
  - Patient returns to seek medical care
  - Patient age (newborn to young infant, < 3 months)
  - Patient with underlying disease
  - Immunocompromised patient
  - Patient underwent recent surgery
  - Patient with an invasive device (venous catheter, urinary catheter, etc.)
  - Patient does not improve or gets worse despite treatment
  - Other

- In the service where you work, how is the diagnosis (ALERT) of SEPSIS/SEPTIC SHOCK made in children? Select more than one alternative, if necessary.
    - We don't have an alert
    - Clinical suspicion by the nursing team
    - Clinical suspicion by the medical team
    - Clinical suspicion by any member of the multidisciplinary team
    - Electronic screening
    - Pediatric Early Warning Signs (PEWS) - early identification alert for clinical deterioration

- In your clinical practice, what clinical criteria do you use to make the early diagnosis of SEPTIC SHOCK?
    - Presence of suspected or confirmed focus
    - Change in temperature (hypothermia or fever)
    - Inappropriate tachycardia
    - Bradycardia for children < 1 year
    - Inappropriate tachypnea
    - Change in capillary refill time
    - Skin color and temperature
    - Pulse amplitude
    - Change in diuresis
    - Change in level of consciousness
    - Hypotension
    - Pulse pressure (SBP minus DBP)
    - Shock index (SBP divided by HR)
    - Other

- In your service, is it possible to obtain rapid venous access (peripheral venous access or intraosseous access - IO) within 5 minutes after suspected diagnosis? Select more than one alternative, if necessary.
    - No, because there are no human and material resources for rapid service.
    - Yes, but only peripheral venous access, as we do not have IO material.
    - Yes, but only peripheral venous access, as we do not have the ability to pass IO
    - Yes, venous and intraosseous access
    - Other

- In your service, is it possible to collect cultures within the first hour after a suspected diagnosis of SEPSIS?
    - We do not have the resources to collect cultures within the first hour after a suspected diagnosis of sepsis
    - Yes, any culture
    - Yes, but only blood culture
    - Other

- In your service, is it possible to administer antimicrobials up to 1 hour after suspected diagnosis of sepsis? Select more than one alternative, if necessary.
    - No, as we do not have antimicrobials available in the unit.
    - No, because the pharmacy takes a long time to release the antimicrobials.
    - No, because I only take antimicrobials after the test results, which take more than 1 hour to be released.
    - No, because there are no human resources for quick service.
    - Yes
    - Yes, but I don't know if this time is controlled.
    - Other

- If you answered YES to the previous question, what is the preferred route of administration you use in your service:
    - Oral route, as we only have oral antibiotics available
    - Oral route, as I do not have the skills or resources to administer antibiotics intramuscularly or intravenously.
    - Intramuscularly, as we do not have the ability or resources to do it intravenously
    - Intravenous route
    - Intraosseous route
    - Other

- In your service, is it possible to administer antimicrobials up to 3 hours after suspected/recognized sepsis? Select more than one alternative, if necessary.
    - No, as we do not have antimicrobials available in the unit.
    - No, because the pharmacy takes a long time to release the antimicrobials.
    - No, because I only take antimicrobials after the test results, which take more than 1 hour to be released.
    - No, because there are no human resources for quick service.
    - Yes
    - Yes, but I don't know if this time is controlled.
    - Other

- In your service, is it possible to administer 40 to 60 mL/kg of fluids within 1 hour after suspected diagnosis? Select more than one alternative, if necessary.
    - No, because we do not have adequate equipment (infusion pump, venous access device, serum)
    - No, because there are no human resources for quick service.
    - No, because I am not afraid of adverse events from volume resuscitation.
    - No, because I do not have the resources to provide care in the event of adverse events from volume resuscitation.
    - Yes
    - Yes, but I'm not sure if that time is controlled
    - Other

- What resuscitation volume rate do you use in your clinical practice to treat children with SEPSIS/SEPTIC SHOCK?
    - 10mL/kg with no weight limit
    - 20mL/kg with no weight limit
    - 10mL/kg maximum 250mL
    - 20mL/kg maximum 500mL
    - Other

- When prescribing fluids for the treatment of children with SEPSIS/SEPTIC SHOCK, do you consider the volume that the patient previously received in another service/location?
    - Never
    - Yes, when fluid infusion was recent (< 6 hours)
    - Yes, when fluid infusion was very recent (< 1 hour)
    - Yes, but only when it was volume resuscitation (fluid bolus)
    - Other

- What solutions for volume resuscitation of children with suspected sepsis do you have in your service? Select more than one alternative, if necessary.
    - Isotonic saline solution
    - Simple ringer
    - Ringer's lactate
    - Plasmalyte
    - Colloid (Albumin)
    - Other

- o Which solutions do you prefer to use (first choice) for volume resuscitation of children with suspected sepsis? Select more than one alternative, if applicable.
    - Isotonic saline solution
    - Simple ringer
    - Ringer's lactate
    - Plasmalyte
    - Colloid (Albumin)
    - Other

- o When do you typically consider infusing a vasoactive amine?
    - In any patient who is already hypotensive/shows signs of hypoperfusion, regardless of the volume received
    - In all patients who do not show improvement after 40 - 60mL/kg
    - I usually wait for evaluation/transfer to ICU
    - Other

- o In your service, is it possible to start vasoactive amines (inotrope/vasopressor) in fluid-refractory shock in the first hour of care? Select more than one alternative, if necessary.
    - No, because we do not have vasoactive amines available.
    - No, because I am not confident in using vasoactive amines.
    - No, because we do not have suitable equipment (infusion pump)
    - No, because there are no human resources for quick service.
    - No, because I do not have the ability to insert a central venous catheter and I do not use vasoactive amines through peripheral access.
    - Yes
    - Yes, but I don't know if this time is controlled.
    - Other

- o What vasoactive amines do you have available in your service? Select more than one alternative, if necessary.
    - Dopamine
    - Dobutamine
    - Adrenaline
    - Noradrenaline
    - Milrinone
    - Arginine-vasopressin
    - Terlipressin
    - Angiotensin
    - Enoximone
    - Levosimendan
    - Other

- o In your department, how is the response to treatment of children with sepsis/septic shock monitored? Select more than one alternative, if necessary.
    - Clinical signs – capillary refill time
    - Clinical signs – pulse characteristics
    - Clinical signs – skin color and temperature
    - Clinical signs – level of consciousness
    - Clinical signs – heart rate
    - Clinical signs – noninvasive blood pressure
    - Urinary output

- Invasive blood pressure
- Central venous pressure
- Continuous monitoring of central venous oxygen saturation
- Intermittent monitoring of central venous oxygen saturation
- Lactate dosage
- Echocardiogram
- USCOM
- PICCO
- Pulmonary artery catheter (Swan-Ganz)
- Other (specify)

o Do you request lactate measurement in cases of clinical suspicion of SEPSIS/SEPTIC SHOCK in children?
- No, because I don't have the exam at work.
- No, because I don't think it's important.
- Yes, in the emergency room.
- Yes, in the pediatric ICU
- Yes, both in the emergency room and in the pediatric ICU
- Other

o In your service, is it possible to monitor central venous oxygen saturation ($ScvO_2$) in suspected SEPSIS/SEPTIC SHOCK in children? Select more than one alternative, if necessary.
- No, because I don't have a central venous catheter at work.
- No, because I am not skilled in inserting a central venous catheter.
- No, because I don't think it's important.
- Yes, in the emergency room.
- Yes, only in the pediatric ICU
- Yes, continuously
- Yes, intermittently
- Other

o If renal replacement therapy is indicated, what is the availability of this therapy in your department? Please indicate more than one alternative, if necessary.
- I do not have availability of renal replacement therapy
- Peritoneal dialysis
- Continuous venovenous hemofiltration (CVVH)
- Continuous venovenous hemodiafiltration (VVCHDF)
- Classical hemodialysis
- Other

o Do you have ECMO available for hemodynamic support in REFRACTORY SEPTIC SHOCK in your service?
- No
- Yes

o Are you available to transfer to an ECMO Center for treatment of REFRACTORY SEPTIC SHOCK?
- No
- Yes

CLINICAL CASES OF SEPSIS / PEDIATRIC SEPTIC SHOCK

- A 10-year-old patient undergoing post-operative spinal arthrodesis. He is febrile (37.9C), HR of 140bmp, broad pulses, warm extremities and capillary refill time < 2 seconds. The BP is 110/50 (70) mmHg, and the RR is 28ipm. Diuresis = 1.5mL/kg/hour. Leukogram = 5,800 cells/mm3. What is the most likely diagnosis?
  - Systemic inflammatory response syndrome
  - Uncomplicated infection
  - Sepsis
  - Severe sepsis
  - Septic shock

- A 6-year-old boy with a 3-day history of fever, cough and fatigue. It appears active, Tax = 38C, HR 144bpm, BP 100 x 50 (65) mmHg, adequate central and peripheral pulses, capillary refill time 2 seconds. RR of 55 ipm with oxygen saturation of 96% in room air, with crackling rales in the left lung. Which are the most probable diagnostics?
  - Systemic inflammatory response syndrome
  - Uncomplicated infection
  - Sepsis
  - Severe sepsis
  - Septic shock

- Girl aged 2 years and 6 months hospitalized with a diagnosis of pneumonia. She is prostrate, febrile (Tax= 38.7C), HR 162bpm, BP 83x44 (57) mmHg, adequate central pulses and fine peripheral pulses, capillary refill time of 4 seconds, RR of 62ipm, oxygen saturation of 92% at 50% Venturia mask. Leukocytosis with more than 10% of young forms. Which are the most probable diagnostics?
  - Systemic inflammatory response syndrome
  - Uncomplicated infection
  - Sepsis
  - Severe sepsis
  - Septic shock

- A 7-year-old boy, with fever, abdominal pain in the right iliac fossa and vomiting for 3 days, without diuresis for 12 hours. He appears prostrate, with dry mucous membranes, febrile, tachycardic, normotensive. He has adequate central pulses and thin peripheral pulses, capillary refill time of 4 seconds, tachypneic. Abdominal ultrasound suggests acute appendicitis. Initial treatment with 0.9% saline solution at 20mL/kg was started. After three expansions (60mL/kg), HR is 166bpm, BP is 80/47 (58) mmHg, capillary refill time is 5 seconds, without diuresis. Which are the most probable diagnostics?
  - Systemic inflammatory response syndrome
  - Uncomplicated infection
  - Sepsis
  - Severe sepsis
  - Septic shock

- 15-year-old girl diagnosed with dysuria. Presents with Tax = 36.7C, HR 88bpm, adequate central and peripheral pulses, capillary refill time of 2 seconds, BP 110 x 65 (80) mmHg, eupneic. Blood leukocytes count of 12,000 cells/mm^3 (no young forms) and presence of leukocyturia. Which are the most probable diagnostics?
  - Systemic inflammatory response syndrome
  - Uncomplicated infection
  - Sepsis
  - Severe sepsis
  - Septic shock
